# Effect of diet supplemented with functional amino acids and polyphenols on gut health in broilers subjected to a corticosterone-induced stress

**DOI:** 10.1038/s41598-023-50852-4

**Published:** 2024-01-10

**Authors:** Sophie Yvon, Martin Beaumont, Alix Dayonnet, Hélène Eutamène, William Lambert, Valérie Tondereau, Tristan Chalvon-Demersay, Pauline Belloir, Charlotte Paës

**Affiliations:** 1 INP-Purpan, Toulouse, France; 2 Toxalim (Research Centre in Food Toxicology), INRAE, ENVT, INP-Purpan, UPS, Université De Toulouse, Toulouse, France; 3 GenPhySE, INRAE, ENVT, Université De Toulouse, Toulouse, France; 4 METEX ANIMAL NUTRITION, Paris, France

**Keywords:** Animal physiology, Nutrition

## Abstract

To address the overuse of antimicrobials in poultry production, new functional feed ingredients, i.e. ingredients with benefits beyond meeting basic nutritional requirements, can play a crucial role thanks to their prophylactic effects. This study evaluated the effects of the supplementation of arginine, threonine and glutamine together with grape polyphenols on the gut integrity and functionality of broilers facing a stress condition. 108 straight-run newly hatched Ross PM3 chicks were kept until 35 days and were allocated to 3 treatments. Broilers in the control group were raised in standard conditions. In experimental groups, birds were administered with corticosterone in drinking water (CORT groups) to impair the global health of the animal and were fed a well-balanced diet supplemented or not with a mix of functional amino acids together with grape extracts (1 g/kg of diet—CORT + MIX group). Gut permeability was significantly increased by corticosterone in non-supplemented birds. This corticosterone-induced stress effect was alleviated in the CORT + MIX group. MIX supplementation attenuated the reduction of crypt depth induced by corticosterone. Mucin 2 and TNF-α gene expression was up-regulated in the CORT + MIX group compared to the CORT group. Caecal microbiota remained similar between the groups. These findings indicate that a balanced diet supplemented with functional AA and polyphenols can help to restore broiler intestinal barrier after a stress exposure.

## Introduction

The gut acts as a sentinel in charge of a controlled uptake of nutrients while preventing the passage of macromolecules and bacteria across the mucosa. These functions can be achieved by a complex crosstalk between the intestinal epithelial cells, the immune system, and the microbiota^1,2^. The poultry caecal microbiota plays a particular role in maintaining gut health and influencing the overall performance with the production of metabolites like short-chain fatty acids, indole, tryptamine, vitamins, and bacteriocins^3^. However, acute and chronic stress can affect this sensitive balance and impair the transport of nutrients^4^, the gut barrier function and the immune response^5^ with persistent effect over time^6^. The disruption of this delicate homeostasis represents a risk factor for digestive diseases^7,8^. Therefore, stressors occurring in broilers farms during breeding, handling and transport of birds can affect flocks’ performances, with variations according to the stress magnitude^9,10^. Innovative solutions are then crucial to mitigate the damaging effects of stress on birds’ health and welfare.

Among the large variety of amino acids (AA), 20 of them are considered as building blocks for protein synthesis. During several decades, nutritional strategies in broilers aimed to use feed-grade free AA associated with low protein diet to reduce feed cost and environmental impact while maintaining performances^11,12^. Besides, findings also suggest that dietary AA could also be used for their regulatory role in metabolism homeostasis, leading to the concept of functional AA^13,14^. Beneficial contributions to gastrointestinal integrity and the immune system can be achieved by increasing specific AA concentrations above the needs for production performance^15^. Functional AA protective effect on the digestive system during enteric challenges is related to their targeted actions on the four pillars of gut health (i.e. oxidative stress, epithelial functions, microbiota and immunity)^16^. The prevalence of intestinal disorders episodes in commercial broilers flocks due to avian coccidiosis (i.e. parasite protozoa *Eimeria*) and enteric viruses, or associated to complex etiologies such as unbalanced diet or environmental troubles (*Salmonella* virus burden, poor building maintenance, heat stress …) is significant in farms and responsible of substantial economic losses for breeders^17^. For instance, a recent study demonstrated that coccidiosis amounted to a cost of £0.16/chicken produced in 2016^18^. Considering the urgent need to reduce drugs uses and environmental disturbs in a one health approach, uses of AA need to be re-thought regarding their metabolic functions and their ability to preserve gut functionalities in commercial broiler farms.

Long considered as anti-nutritional factors, the use of bioactive compounds from plant such as polyphenols is also gaining increasing attention to promote a better intestinal health^19^. Polyphenols encompass a large diversity of raw materials and bioactive compounds, from simple to complex molecules (tannins, flavonoids…), resulting in different physiological outputs. Polyphenols have been showed to exert antibacterial, antioxidant, immunostimulatory and prebiotic properties^20,21^. Grape products in particular attracts professional of animal nutrition due to their high contents of flavonoids and phenols. For instance, a sustained administration of grape by-products in broilers feed were shown to preserve the antioxidant/oxidant balance of birds infected with *Eimeria tenella*^22^. At adequate doses, the use of plant extracts in broiler nutrition can successfully improve the broiler gut health status^23,24^, especially when used in combinations^25^.

Combining compounds targeting different aspects of the gut health not only allows to preserve intestinal functions but can also contribute to reduce inclusion rates of active compounds through additive effects and/or synergies. Previous studies demonstrated the benefits of adding grape extract polyphenols to a mix of L-Arg, L-Thr and L-Gln in terms of gut permeability^26^ and amino acid ileal digestibility^27^ for broilers under stress. In particular, the use of AA in combination with polyphenols might represent an effective association because of (i) their separate beneficial effects on the gut microbiota and epithelium^28^ and (ii) through the binding of AA to tannins, ensuring a protection to AA degradation in the upper side of the digestive tract^29^. Thereby, a supplementation of arginine (Arg), glutamine (Gln) and threonine (Thr) together with grape extract (combination provided at 0.1%) was shown to reverse gut leakage and inflammation under glucocorticoid challenge^26^ and coccidian challenge^30^ and was equally effective than the inclusion of Arg alone at high level (0.5%)^26^. Because broilers experience higher needs for functional AA when facing intestinal challenges^16,31^, identifying nutritional solutions to optimize AA benefits is of particular interest. In this study, we therefore aimed to examine the potential of an amino acids and polyphenols mix for broilers to cope with a stress induced by the administration of corticosterone (CORT), the primary corticosteroid in birds produced during the stress hormonal cascade^32^. Corticosterone was provided via drinking water, as a non-invasive stress model for poultry^33^, able to disrupt the epithelial barrier by elevating plasma CORT^34,35^. Intestinal health is a complex trait and its assessment requires multiple approaches and biomarkers^36,37^. Gut microbiota composition changes with chicken age, genotype, and production system^3^. Therefore, we propose herein a comprehensive gut health assessment by investigating at two developmental points, the starting phase (14–16 days of age) and the growing phase (32–35 days of age), gut microbiota profile and metabolites and different features of the intestinal wall, i.e., the intestinal paracellular permeability reflecting gut integrity, the epithelium morphology and the inflammatory response of the intestine.

## Material and methods

### Ethics declaration

All animal experiments were approved by the French ethics committee n°115 (file reference: #28690). Chickens were raised and handled at Lamothe Farm in the broiler experimental station (Ecole d’Ingénieurs de PURPAN, Seysses, France) according to the European Union’s guidelines and regulations concerning the protection of animals used for scientific purposes (2010/63/EU). Reporting in the manuscript follows recommendations in ARRIVE guidelines.

### Husbandry and experimental design

Straight-run newly hatched Ross chicks (PM3) vaccinated against Marek's disease were purchased from a local hatchery (SOCAVIC, Monferran-Savès, France) and raised from day 1 (d1) to d35 in our experimental poultry unit. A controlled age-appropriate environment was provided to the animals according to AVIAGEN guidelines^38^. The ambient temperature program applied was 31 °C from d1 to d3, 29 °C from d4 to d6, 28 °C from d7 to d9, 26 °C from d9 to d16, 24 °C from d17 to d20, 22 °C from d21 to d27 and 20 °C from d28 until the end of the experiment. Birds were exposed to the following light schedule: 23 h of light from d1 to d3, 20 h of light at d4 and 18 h of light from d5 to d35.

Chickens had unrestricted access to feed and water, except 12 h before intestinal permeability evaluation at d14 and d32. No antibiotic treatment was administered during the experiment.

**Figure 1:**
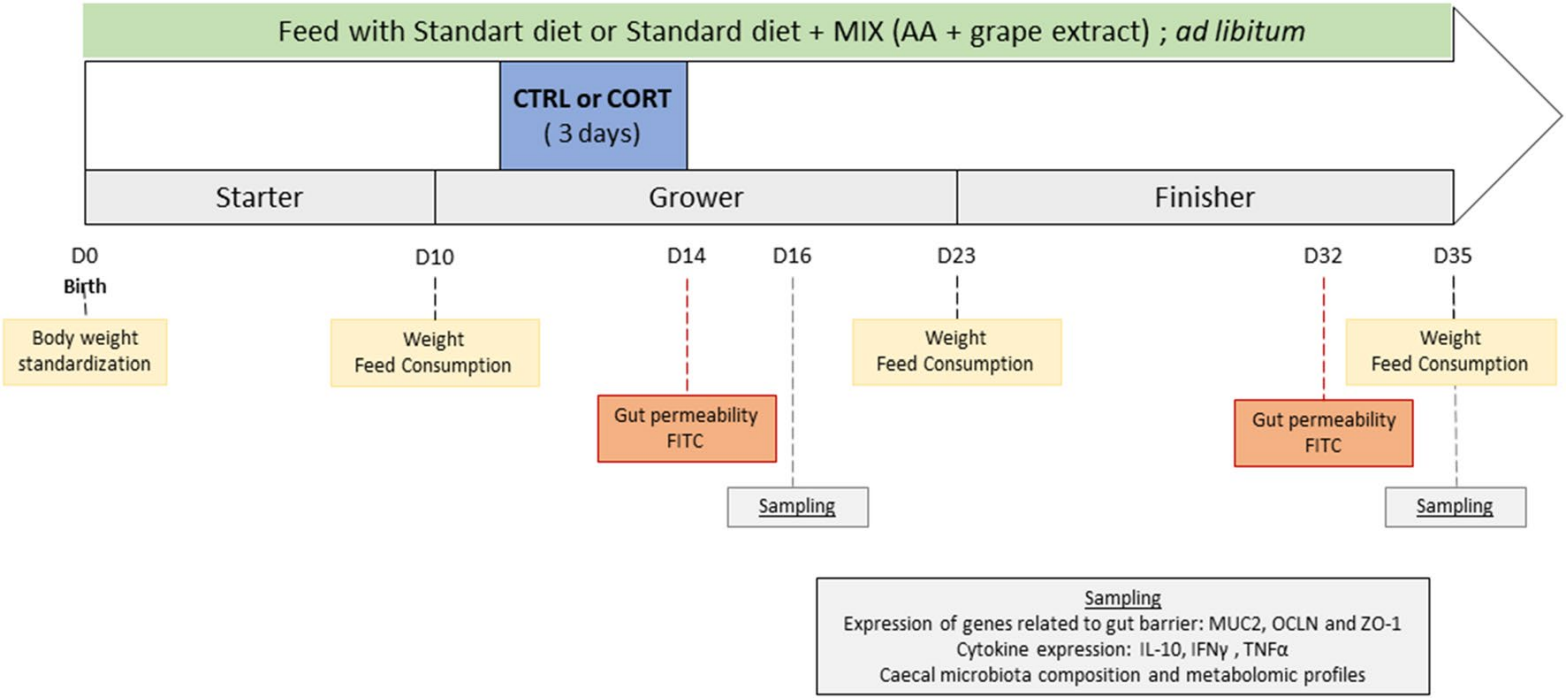
Experimental design of the study. CTRL, control group; CORT, birds challenged; CORT + MIX, birds challenged and supplemented with an amino-acid and polyphenols solution.

At d1, a total of 108 Ross PM3 birds of similar weight (40 ± 2 g) were distributed into 12 furnished cages of 1 m^2^ (n = 9 chicks per cage). They were randomly allotted to 3 experimental groups (4 cages/group): standard diet (CTRL), standard diet with a short-term corticosterone treatment (CORT), standard diet supplemented at 1 g per kg of pelleted feed with functional AA together with grape extract polyphenols and with a short-term corticosterone treatment (CORT + MIX) (Fig. 1). The grape extract was obtained from seeds and skins (85% and 15% respectively) and contains around 70% of polyphenols on dry material. Proanthocyanidins, a class of polyphenols from the favonoid family, are the predominant polyphenols in the grape extract used. This AA and polyphenols blend has been previously tested and reported in different conditions^27,28,39^.

Corticosterone (CORT) was used to mimic in a reproducible manner the effects of hormone-related stress as previously described in avian species^34,35^. CORT (Sigma, Aldrich, MO) was dissolved in ethanol and diluted in tap water at a concentration of 20 mg/L, a dose capable of elevating plasma CORT^34^. Short-term CORT challenge was applied to the birds of groups CORT and CORT + MIX via drinking water for 3 days, starting from d11, with a replacement of the solution every morning. Birds from CTRL group had access to a specific drinker line without CORT.

### Experimental diets

Feeding was done in 3 phases: starter (d1–d10), grower (d10–d23), and finisher phases (d23–d35). All the diets were steamed pelleted and distributed to broilers ad libitum except before intestinal permeability evaluation*.* The basal diet was formulated with common raw materials to meet the broilers nutritional requirements according to Belloir et al.^11^ (Table 1). Diets were analyzed for dry matter (AFNOR methods, V18-109), N contents (ISO 16634-1, Kjeldahl method) and AA content (ISO 13903) by Metex NOOVISTAGO laboratory (Amiens, France).

**Table 1:** Ingredients and chemical composition of the standard diet in period of starter (d1–d10), grower (d10–d23) and finisher (d23–d35).

	Starter (d1–d10)	Grower (d10–d23)	Finisher (d23–d35)
Ingredients (g/kg as fed-basis)			
Corn	380	400	400
Soybean meal (48% CP)	339	302	276
Wheat	200	212	229
Oil, soybean	36	46	58
Monocalcium phosphate	15.0	12.5	10.9
Calcium carbonate	11.8	10.8	9.4
Sodium bicarbonate	1.3	1.3	1.5
Salt	3.0	3.0	3.0
DL-MET 99%	3.7	3.2	2.9
L-LYS HCL 99%	2.6	2.4	2.3
L-THR 98.5%	1.5	1.4	1.2
L-VAL 98%	1.0	0.8	0.7
Vitamin-minerals premix^a^	0.5	0.5	0.5
Phytase 0.006%	0.1	0.1	0.1
Phytase 0.005%	0.1	0.1	0.1
Calculated composition (%)			
Dry matter	89.5	89.9	90.6
CP	22.2	20.5	19.3
AME (kcal/kg)^b^	2950	3050	3150
Tot LYS	1.37	1.25	1.20
Tot MET	0.62	0.56	0.54
Tot MET + CYS	0.96	0.88	0.84
Tot TRP	0.28	0.25	0.24
Tot THR	0.93	0.86	0.80
Tot LEU	1.71	1.58	1.50
Tot ILE	0.95	0.86	0.80
Tot VAL	1.09	0.99	0.93
Tot ARG	1.41	1.28	1.21
Tot HIS	0.54	0.50	0.47
Tot PHE	1.09	0.99	0.93
Tot TYR	0.77	0.69	0.65
Digestible LYS	1.20	1.10	1.03
Digestible MET	0.64	0.58	0.53
Digestible MET + CYS	0.90	0.82	0.77
Digestible TRP	0.24	0.22	0.21
Digestible THR	0.80	0.74	0.69
Digestible LEU	1.52	1.42	1.35
Digestible ILE	0.80	0.74	0.70
Digestible VAL	0.96	0.88	0.82
Digestible ARG	1.26	1.15	1.08
Digestible HIS	0.47	0.43	0.41
Digestible PHE	0.93	0.86	0.82
Digestible TYR	0.67	0.62	0.58
Analysed composition (%)			
CP	22.0	20.4	19.3
AME (kcal/kg)^b^	2950.00	3050.01	3150.10
Tot LYS	1.34	1.23	1.15
Tot MET	0.67	0.61	0.56
Tot MET + CYS	1.01	0.93	0.88
Tot TRP	0.27	0.25	0.23
Tot THR	0.95	0.87	0.82
Tot LEU	1.75	1.64	1.55
Tot ILE	0.93	0.85	0.80
Tot VAL	1.11	1.02	0.95
Tot ARG	1.42	1.31	1.22
Tot HIS	0.54	0.51	0.48
Tot PHE	1.08	1.00	0.94
Tot TYR	1.34	1.23	1.15

^a^Retinyl acetate, 15,000 IU; cholecalciferol, 5000 IU; dl-α tocopherol acetate, 100 mg; menadione, 5 mg; thiamine, 5 mg; riboflavin, 8 mg; pyridoxine, 7 mg; cyanocobalamin, 0.02 mg; niacin, 100 mg; folic acid, 3 mg; biotin, 0.3 mg; calcium pantothenate, 25 mg; choline, 550 mg; manganese oxide, 80 mg; zinc sulfate, 90 mg; iron sulfate, 50 mg; copper sulfate, 20 mg; calcium iodine, 2 mg; sodium selenium, 0.2 mg; cobalt carbonate, 0.6 mg; butylated hydroxytoluene, 125 mg; Salt: 0.212%; Sodium bicarbonate: 0.12%; Monteban: 0.05%.

^b^Calculated Apparent Metabolisable Energy according to Sauvant et al. (2004^70^).

A mix of functional AA (L-Arg, L-Thr, L-Gln) and grape seed and skin extract (GSE) (INNEUS®, Metex NOOVISTAGO) was provided to CORT + MIX group at 1 g per kg of pelleted feed.

### Sampling

At d16 and d35, two broilers per pen, representative of the average BW in the pen, were selected (8 broilers/treatment), weighed and euthanized by electronarcosis followed by exsanguination. Immediately after euthanasia, a segment of mid jejunum was removed and divided into three sections. One section was immerged in RNA*later* solution (ThermoFisher Scientific, Waltham, MA) followed by a storage at − 80 °C until RNA isolation. The second section was rinced in NaCl (0.9%), fixed by immersion in phosphate-buffered formalin (10%) and stored in ethanol 70% (v/v) until histomorphometry analysis. The third jejunum section was fixed in Carnoy buffer (60% ethanol, 30% chloroform, 10% glacial acetic acid; Carnoy was used for optimal preservation of the mucus layer in tissue samples), dehydrated, and embedded in paraffin following a standard protocol^40^. For the microbiota and metabolome analyses, caecal digesta was collected in sterile conditions before storage at − 80 °C.

### Gut permeability measurement

In vivo paracellular permeability was assessed by measuring the amount of fluorescently labelled dextrans (FITC-d) that had crossed the digestive epithelium into the blood after oral gavage. Elevated blood levels of this large-size molecule are recognized as an indicator of disrupted tight junctions (TJ)^7^. According to the animal developmental point, two (d14) or three (d32) chickens per cage subjected to 12 h of feed restriction were randomly selected and orally received FITC-dextran solution (3–5 kDa FITC-d dissolved in saline solution; Sigma Aldrich). An appropriate dose of 8.32 mg/kg of body weight was given as recommended in broiler model^41^. Blood samples were collected 1 h after FITC-d administration in heparin tubes at the brachial wing vein (n = 8 chickens/treatment at d14 and n = 12 at d32) and temporary stored in a dark-colored container. After centrifugation (1000 g for 10 min at 4 °C), the plasma were collected and diluted in phosphate buffer saline (at 1:2 or 1:6 according to the sample concentration). Plasma FITC-d levels were determined by fluorometric mesureament on the same day (excitation, 485 nm; emission 528 nm; SPARK; TECAN, Männedorf, Switzerland) based on a calculated standard curve (saline solution as a blank).

### Histological analysis

#### Histomorphometry

The method used to assess the jejunum tissue morphology was based on the one described by Goodlad et al.^42^. Formalin-fixed biopsy samples were used instead of paraffin-embedded ones to avoid retraction artefacts of paraffin-embedded tissue. The jejunum tissue was transferred from ethanol to a mixture (1:3) of acid acetic (45%) and ethanol (70%) for at least 12 h. Afterwards, the tissue was rehydrated in a bath of ethanol/water (1:2) and then placed in distilled water. Thereafter, the samples were stained with the Feulgen reaction: hydrolysis in 1N hydrochloric acid at 60 °C for 6 min, rinsing three times with distilled water, and staining with Schiff’s reagent for 30 min (Sigma-Aldrich, Darmstadt, Germany). Villus and crypts were dissected and isolated from the connective tissue under a binocular magnifier using fine-gauge syringe. For each animal (n = 7–8/group), an average of 10 villus and 20 crypts were randomly selected. The preparation was mounted on glass slide in a drop of aqueous mounting media (Aquatex; Merck, Darmstadt, Germany). Under an optical microscope (Leica DM750, Wetzlar, Germany) equipped with a camera (CMEX5000; Euromex, Arnhem, Netherlands), villus height, villus width, crypt depth and crypt width were measured (based on the maximum for the width) with a magnification of 40 and 100 by using the image analyser ImageFocus® (Euromex).

#### Evaluation of goblet cells density

Jejunum tissues embedded in paraffin blocks were cut perpendicular to the axis of the intestine into thin sections (4 µm) on a microtome (HM340E; ThermoFisher Scientific). Sections were mounted on microscope slides (Thermo Scientific Menzel-Gläser Superfrost® Plus slides) and then stained with a combination of periodic acid Schiff (PAS, Sigma-Aldrich) for neutral mucins and Alcian Blue 8GX at pH 2.5 (Biognost, Wevelgem, Belgium) for acid mucins. Sections from the same sampling day (n = 8/group) were stained in one batch. Mucin-containing cells (goblet cells) were counted in 10 full-length villi. The height and width of these well-oriented villi were measured in parallel. The density of goblet cells per unit area was finally determined by dividing the absolute number of goblet cells by the corresponding villus area. The same microscope, digital camera and analysis program of the histomorphological protocol were used.

### Profiling of jejunum gene expression

Total RNA in the jejunum tissue was isolated using the Direct-zol RNA MiniPrep Plus kit (ZymoResearch, Irvine, CA) according to the manufacturer instructions. RNA quantity and quality (control of 260/280 and 260/230 ratios) were determined using NanoDrop-2000 (Thermo Fisher Scientific). cDNA were prepared from 1 µg RNA with Superscript II (ThermoFisher Scientific) in the presence of random primers and RNase inhibitor following the manufacturer recommendations. After a dilution at 1:10, quantitative PCR were carried in triplicate on cDNA using the thermal cycler C1000 Touch™ (Bio-Rad, Hercules, CA). The cycling conditions consisted of a denaturation step (95 °C for 10 min) and 40 cycles of amplification, including both denaturation for 15 s at 95 °C and annealing-extension for 1 min at 60 °C. At the end of the PCR, dissociation was carried out by slowly heating the samples from 60 to 95 °C and continuous recording of the decrease in SYBR Green fluorescence. Primers used in this study were related to gut integrity and immunity and were sourced from literature (Supplemental Table 1). Primers were checked for efficiency and specificity using melting curve analysis. The relative fold changes of target genes expression were calculated with the 2^−ΔΔCt^ method with GAPDH as the housekeeping gene and CTRL group at d16 as the reference.

### 16S rRNA gene amplicon sequencing and sequence analysis

Total DNA was extracted using the Quick-DNA Fecal/Soil Microbe 96 Kit (ZymoResearch) and the 16S rDNA V3-V4 region was amplified by PCR and sequenced by MiSeq Illumina Sequencing as previously described^43^. 16S rRNA gene amplicon sequences were analyzed using the FROGS pipeline according to standard operating procedures^44^. Amplicons were filtered according to their size (350–500 nucleotides) and clustered into OTUs using Swarm (aggregation distance of 3). After chimera removal, OTUs were kept when present in at least 3 samples or representing more than 0.005% of the total number of sequences^45^. OTUs affiliation was performed using the reference database silva138 16S with a minimum pintail quality of 100^46^. The mean number of reads after filtering per sample was 19 720 (min: 15 514–max: 22,864).

### NMR metabolomics

Caecal metabolome was analyzed by using nuclear magnetic resonance (NMR) based metabolomics as described before^47^. Briefly, metabolites were extracted from 50 mg caecal content in a phosphate buffer prepared in D_2_O. The samples were transferred in 5 mm NMR tubes and analyzed by the Carr-Purcell-Meiboom-Gill spin-echo pulse sequence with an Avance III HD NMR spectrometer operating at 600.13 MHz for ^1^H resonance frequency using a 5 mm inverse detection CryoProbe (Bruker Biospin, Rheinstetten, Germany). The spectra were processed with the R package ASICS for baseline correction, bucketing (0.01 ppm) and normalization by the total area^48^. For metabolite identification, spectra of pure compounds prepared in the same buffer and acquired with the same spectrometer were overlayed with sample spectra. For each identified metabolite, buckets non-overlapping with other metabolites were selected for the quantification (Supplemental Table 2).

### Data analysis

All statistical analyses were performed using R software (version 4.0). Value *p* < 0.05 was considered significant all along the study. Levene's tests were performed to check for equality of variances before parametric analyses. False discovery rate adjustments were used for multiple testing.

Log-transformed gene expression data was subjected to two-way ANOVA to assess the effect of the treatment, age, and their interaction. A Box-Cox transformation was applied on FITC-d data to meet the homogeneity assumption. Goblet cell count data and morphometric data were log transformed for normality. Linear mixed procedure of nlme package was used to analyze BW longitudinal data, FITC-d concentrations and morphometric data with the treatment and age as fixed effects, the cage as random effect and a correction for age heteroscedasticity. To consider the variability of paraffin-embedded sections, mixed model was applied on goblet cell measurement with the experimental treatment and age as fixed effect and the histological section as a random variable.

The microbiota composition analysis was performed using the phyloseq package^49^. Within-community diversity was evaluated after rarefaction of the OTU table at 15 501 sequences. To examine differences in community structure, Bray–Curtis, UniFrac and wUniFrac distances were calculated on the rarefied matrix. A PERMANOVA was then used to perform pairwise comparison between groups. Two-way ANOVA was used to analyze metabolites relative concentrations (log-transformed) and bacterial relative abundances at the phylum, family and genus levels (fourth root transformed) after discarding the OTU representing less than 0.05% of the total number of sequences.

## Results

### Broilers body weight evolution

Animals euthanized from the control group weighed 616 ± 100 g at d16 and 2210 ± 241 g at d35 (n = 8), in accordance with AVIAGEN performance objectives^38^. Corticosterone administration reduced the birds body weight at d16 in the CORT group (486 ± 40 g; *p* = 0.02) and in the CORT + MIX group (516 ± 81 g; *p* = 0.03), compared to the control group (n = 8 euthanized broilers /group). No difference was observed in broilers body weight between the CORT and CORT + MIX groups at this age. At d35, no significant difference in body weight between the birds sampled was observed.

Supplementary Table 3 shows the performance results when considering the entire flock. At d10, the birds in the CORT group were significantly lighter (*p* < 0.001). Thereafter, the growth performance of the broilers was similar between the groups.

### Intestinal permeability and integrity

**Figure 2:**
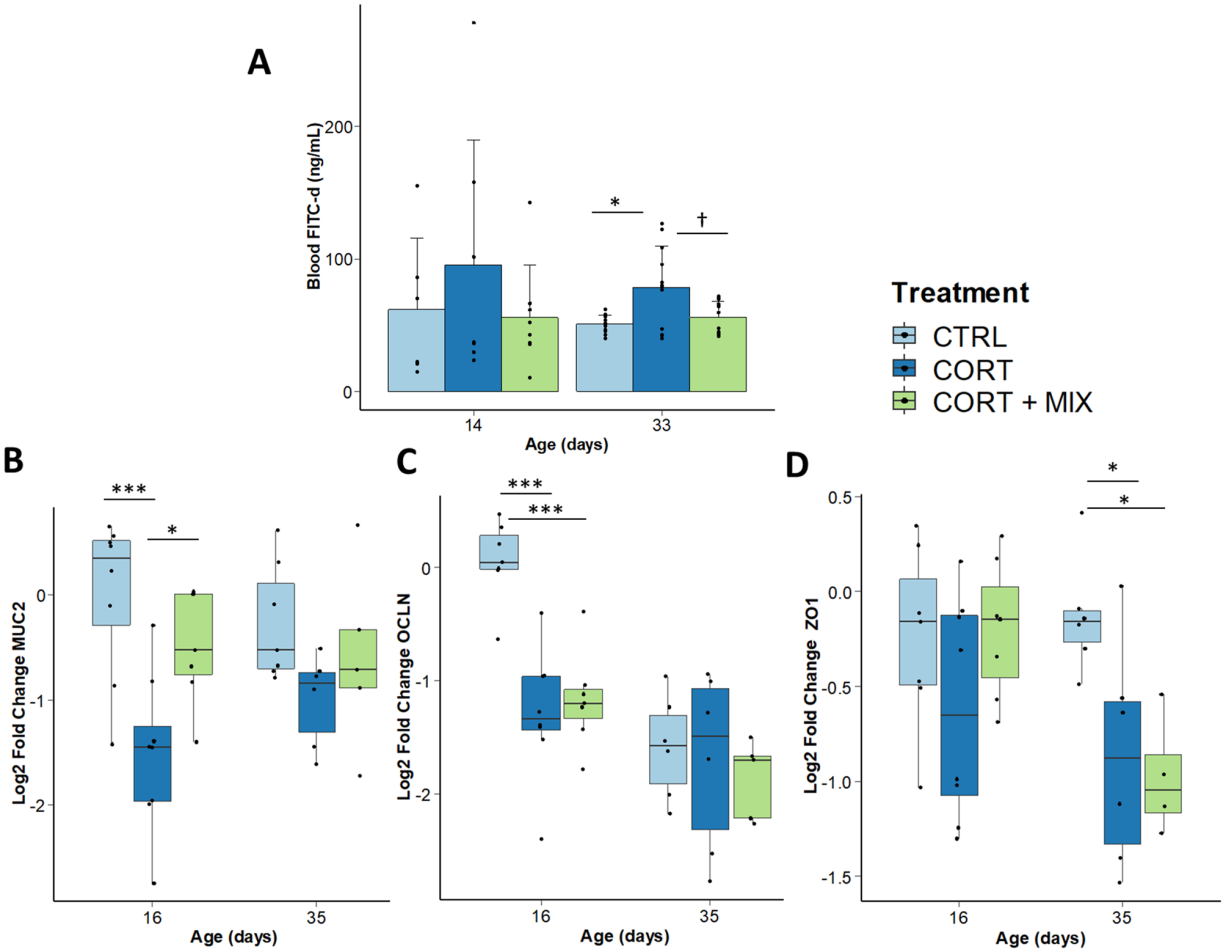
Assessment of gut barrier integrity after a corticosterone challenge and the provision of a nutritional solution containing functional amino acids and polyphenols. (**A**) Fluorescein isothiocyanate dextran (FITC-d) plasma levels of control and treated chickens (3–5 kDa FITC-dextran gavaged at 8.32 mg/kg, n = 8 chickens/treatment at d14 and n = 12 at d32). Error bars stand for standard deviation. (**B**–**D**) Jejunum mRNA levels of gene expression at d16 and d35. Relative mRNA levels were measured by real time RT-PCR (n = 8 chickens/treatment/age). B: MUC2 (mucin 2); C: OCLN (occludin); D: ZO1 (Tight junction protein-1). Significant differences between treatments are reported (†: *p* < 0.1, *: *p* < 0.05, **: *p* < 0.01, ***: *p* < 0.001). CTRL, control group; CORT, birds challenged; CORT + MIX, birds challenged and supplemented with an amino-acid and polyphenols solution.

The levels of FITC-d measured in plasma was used as an indicator of the integrity of the whole gastrointestinal tract (Fig. 2A). At d14, no significant difference was observed between the experimental groups although the plasma FITC-d values of the CORT group were numerically higher. The high variability observed at this sampling time (70 ± 66 ng/mL of FITC-d) justified to increase the sampling size at the second time of sampling (+ 4 animals/group). At d33, the FITC-d variance was higher in the CORT group compared to the others (*p* = 0.03). After a correction for heteroscedasticity, we showed that CORT challenge without MIX supplementation increased the concentration of FITC-d during the finisher phase (+ 50% when compared with CTRL group, *p* = 0.05), indicating higher paracellular transport across the gut epithelium after glucocorticoid administration. In the CORT + MIX group, the levels of FITC-d at d33 were non-statistically different from the control animals and tended to be lower than in CORT broilers (*p* = 0.07).

To further understand the mode of action of the MIX supplementation in this context of gut barrier dysfunction, the mucus layer and the intercellular junctional complexes of the epithelial cell layer were studied as two crucial components of the gut integrity. Relative jejunal expression of genes involved in these two lines of defense are presented in Fig. 2B. CORT challenge in the absence of supplementation decreased mucin 2 (*MUC2*) expression compared with the control group (*p* < 0.001) and the supplemented birds (*p* = 0.01) at d16. During the finisher phase, no modulation of *MUC2* expression was observed. The density of goblet cells, specialized for the synthesis and secretion of mucus, was then assessed in the villi (Table 2). The goblet cell density in birds of the CTRL group was significantly higher than in the CORT + MIX group at d16 (+ 15 cells/villi), while no effect of the treatments was observed at d35. Expression of the tight junction protein Occludin (*OCLN*) was downregulated in CORT and CORT + MIX groups compared with CTRL (*p* < 0.001) at d16. A significant decrease of *OCLN* mRNA levels was then observed between d16 and d35 in CTRL and CORT + MIX groups. The expression of the tight junction protein Zonula occludens-1 (*ZO-1*) was affected by the CORT challenge at d35 with a significant decrease in CORT and CORT + MIX groups when compared with CTRL (*p* = 0.02).

**Table 2:** Goblet cell density of birds challenged or unchallenged with corticosterone and fed a standard diet or supplemented with a nutritional solution containing functional amino acids and polyphenol.

	CTRL		CORT		CORT + MIX		*p* value		
	d16	d35	d16	d35	d16	d35	Treatment	Age	Treatment × age
Goblet cell density (cell counts per mm^2^)	515 ± 154^b^	522 ± 176	426 ± 159^ab^	534 ± 231	408 ± 160^a^	422 ± 155	0.03	0.20	0.48
Goblet cell density (cell counts per villi)	61 ± 21^b^	84 ± 34	52 ± 20^ab^	73 ± 36	46 ± 18^a^	86 ± 37	0.1	***	0.09

Neutral and acid mucins were measured by staining sections with Alcian blue and PAS. Goblet cells were taken from 10 villi per bird (n = 8 chickens/treatment/age). Values are means ± SD. Means with no common superscripts differ significantly (*p* < 0.05). *** : *p* < 0.001.

### Evaluation of the epithelium architecture

The intestinal morphology was evaluated by in situ measure of villous height and crypt depth on micro dissected samples (Table 3). Upon aging, the intestine architecture was modified with increased villi size between the grower and finisher phases (*p* < 0.001). At d16, the birds of the CORT + MIX group showed the smallest villi surface (− 32% and − 28% when compared with CTRL and CORT group; *p* < 0.001), because of lower villus height and width. At d35, a reduction of villus width was observed in birds of the CORT group when compared with the CTRL broilers (*p* = 0.05) and CORT + MIX (tendency; *p* = 0.09). While no treatment effect was reported at d16 regarding crypt morphology, crypt depth and surface were lower in the CORT group compared with CTRL and CORT + MIX birds at d35 (Table 3). In summary, at day 16, villus morphology from CORT group was no altered, while animals from CORT + MIX group exhibited smaller villus size. CORT induced adverse effects on crypt structure at d35, which were counteracted with MIX supplementation.

**Table 3:** Morphology measurement of jejunum villi and crypts of broilers fed with experimental diets at d16 and d35.

	CTRL		CORT		CORT + MIX		*p* value		
	d16	d35	d16	d35	d16	d35	Treatment	Age	Treatment × age
Villus height (mm)	0.73 ± 0.15^b^	0.99 ± 0.13	0.75 ± 0.13^b^	1.08 ± 0.20	0.60 ± 13^a^	0.99 ± 0.18	0.06	***	***
Villus width (mm)	0.69 ± 0.14^b^	0.92 ± 0.23^B^	0.63 ± 0.12^ab^	0.74 ± 0.14^A^	0.57 ± 0.13^a^	0.87 ± 0.18^AB^	0.10	***	***
Villus surface (mm^2^)	1.02 ± 0.33^b^	1.84 ± 0.59	0.96 ± 0.30^b^	1.63 ± 0.50	0.68 ± 0.24^a^	1.69 ± 0.33	0.04	***	***
Crypt depth (mm)	0.16 ± 0.03	0.20 ± 0.04^B^	0.18 ± 0.04	0.14 ± 0.03^A^	0.18 ± 0.06	0.18 ± 0.04^B^	0.40	0.59	***
Crypt width (mm)	0.06 ± 0.02	0.06 ± 0.02	0.06 ± 0.01	0.06 ± 0.01	0.07 ± 0.02	0.06 ± 0.02	0.45	0.05	0.06
Crypt surface (mm^2^)	0.021 ± 0.007	0.024 ± 0.008^B^	0.023 ± 0.008	0.0018 ± 0.005^A^	0.025 ± 0.012	0.022 ± 0.008^B^	0.25	0.04	***

Epithelium morphometry was evaluated by microdissection after Schiff staining. 10 villi and 20 crypts per bird were evaluated (n = 8 chickens/treatment/age). Values are means ± SD. Means with no common superscripts differ significantly within one specific age (*p* < 0.05). *** : *p* < 0.001.

### Cytokines gene expression

**Figure 3:**
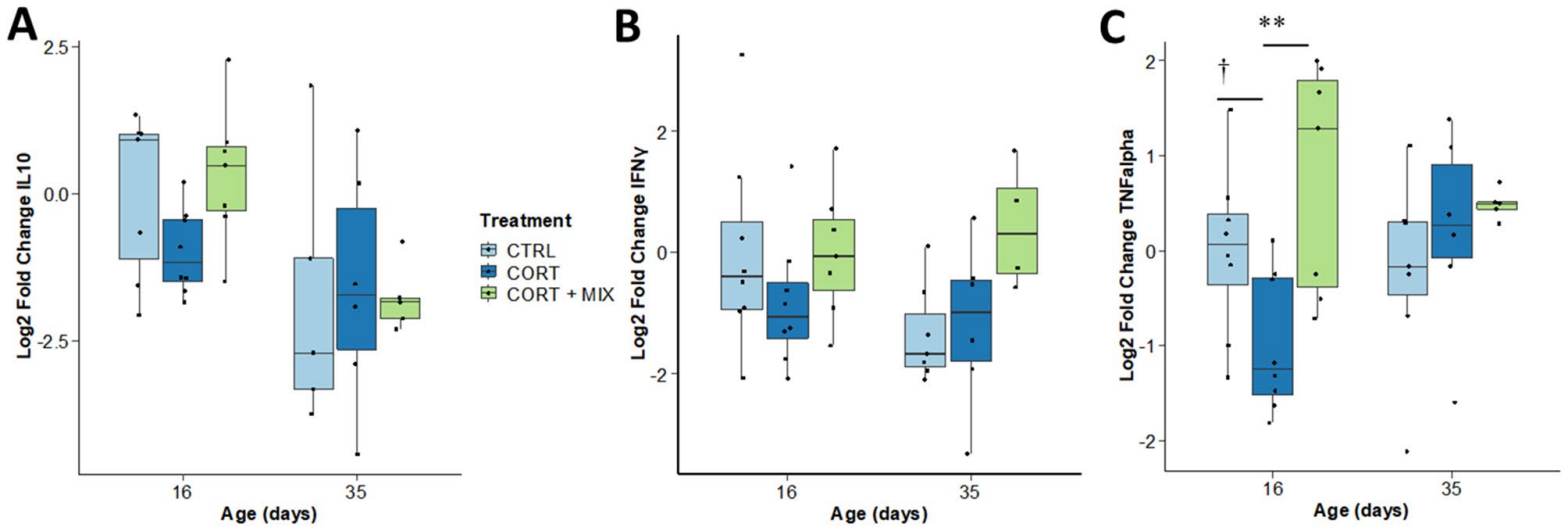
Cytokine gene expression in the jejunum. Relative mRNA levels were obtained by real time RT-PCR (n = 8 chickens/treatment/age). Significant differences between treatments are reported (†: *p* < 0.1, **: *p* < 0.01). Error bars stand for standard deviation. CTRL, control group; CORT, birds challenged; CORT + MIX, birds challenged and supplemented with an amino-acid and polyphenols solution. A: *IL10* (interleukin 10); B: *INFγ* (interferon gamma); C: *TNFα* (tumor necrosis factor alpha).

Knowing the key role of the cytokines in intestinal immunity, the expression of three cytokines was analyzed (Fig. 3). The expression of the potent anti-inflammatory cytokine *IL-10* decreased over time (*p* = 0.003), but mRNA levels were not affected by the experimental conditions. The expression of *IFNγ* was unaffected by the age of the animals and their treatment groups. The expression of the cytokine *TNFα* was up-regulated in CORT + MIX group when compared with CORT group at d16 (*p* < 0.01).

### Caecal ecosystem: microbiota composition and metabolomic profiles

Because the gut microbiota can mediate the effects of feed components on the gut health, the structure and composition of the caecum microbiota was analyzed. The species richness in the caecum was increased with age but was not affected by the CORT challenge nor the supplementation at the different time points of the analysis. Similarly, the treatments did not affect the Shannon and InvSimpson alpha-diversity indexes at d16 and d35 (Table 4).

**Table 4:** Alpha-diversity in cecal microbiota of broilers challenged or unchallenged with corticosterone and fed a standard diet or supplemented with a nutritional solution containing functional amino acids and polyphenol.

	CTRL		CORT		CORT + MIX		*p* value		
	d16	d35	d16	d35	d16	d35	Treatment	Age	Treatment × age
Number of observed OTUs	230.6 ± 21.8	308.8 ± 15.2	233.8 ± 28.4	299.4 ± 21.9	243.6 ± 30.2	313.8 ± 20.5	0.24	***	0.66
Shannon index	3.2 ± 0.5	3.4 ± 0.5	3.7 ± 0.2	3.4 ± 0.3	3.6 ± 0.2	3.5 ± 0.4	0.12	0.50	0.25
InvSimpson index	10.4 ± 5.7	11.8 ± 7.5	18.1 ± 4.9	10.3 ± 4.8	16.1 ± 5.5	10.6 ± 6.8	0.19	0.04	0.10

Values are means ± SD (n = 8 chickens/treatment/age). Means with no common superscripts differ significantly within one specific age (*p* < 0.05). *** : *p* < 0.001.

**Figure 4:**
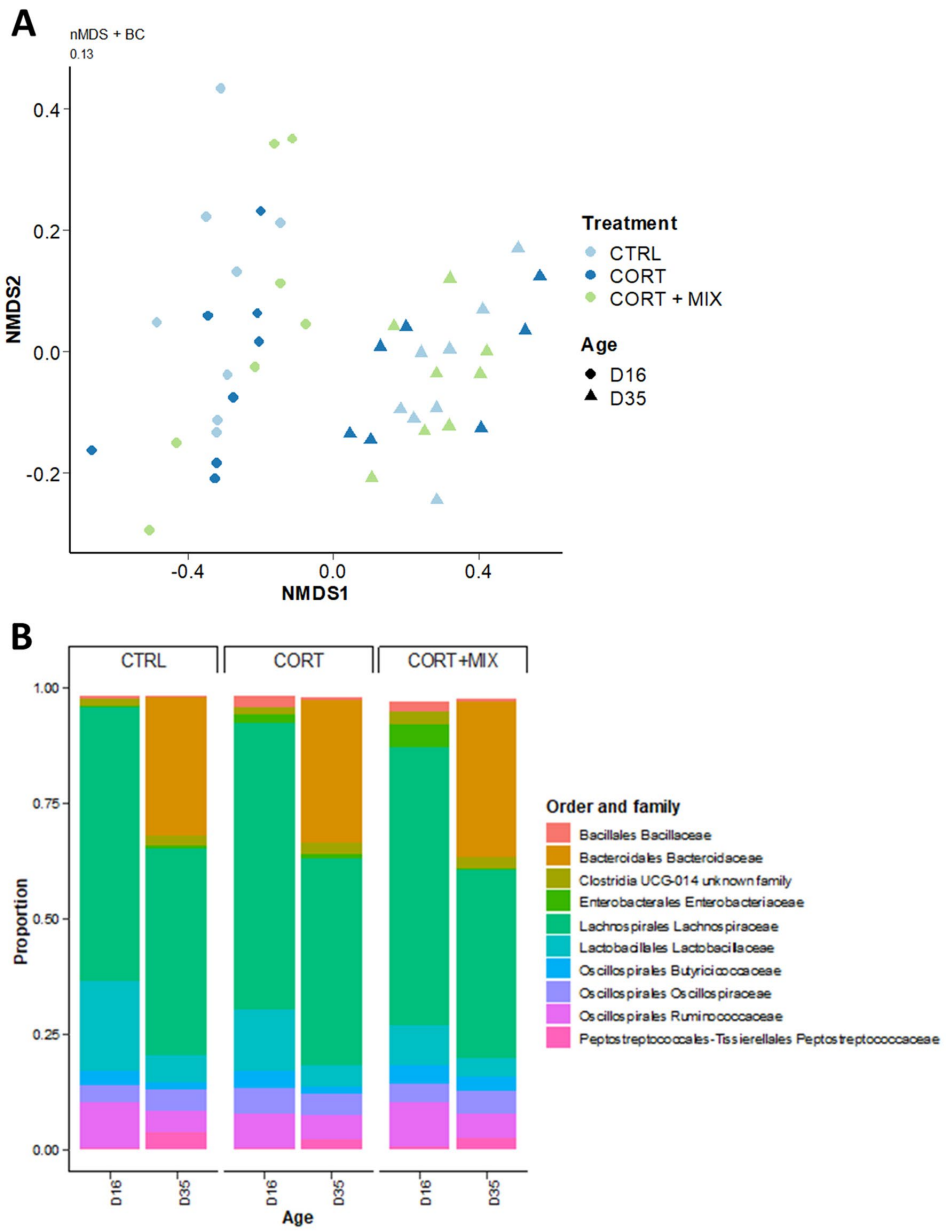
(**A**) Non-Metric Dimensional Scaling (nMDS) two-dimensional representation of cecal bacterial community using Bray Curtis distances of chickens in groups CTRL, CORT, and CORT + MIX at d16 and d35. (**B**) Distribution of the 10 most abundant families in the caeca of broilers from different groups. *CTRL* Control group, *CORT* Birds challenged, *CORT* + MIX, birds challenged and supplemented with an amino-acid and polyphenols solution.

The dissimilarities between the caecal bacterial communities were evaluated with Bray–Curtis, UniFrac and wUniFrac distances. Pairwise comparison did not exhibit different distance indices between the treatments, while the age explained between 31 and 48% of the variations in the calculated distances (*p* < 0.001). The nMDS projection of the microbial communities with Bray distances (Fig. 4) confirmed the absence of clustering between CTRL, CORT and CORT + MIX groups. The 16S rRNA taxonomic microbial profiling was then assessed at the different taxonomic levels. *Firmicutes* represented the most predominant phyla by accounting for 98% and 70% of total relative abundances at d16 and d35, with no difference between the experimental groups. The proportions of families with relative abundances superior to 0.5% were not statistically different between the treatments (Fig. 4).

**Figure 5:**
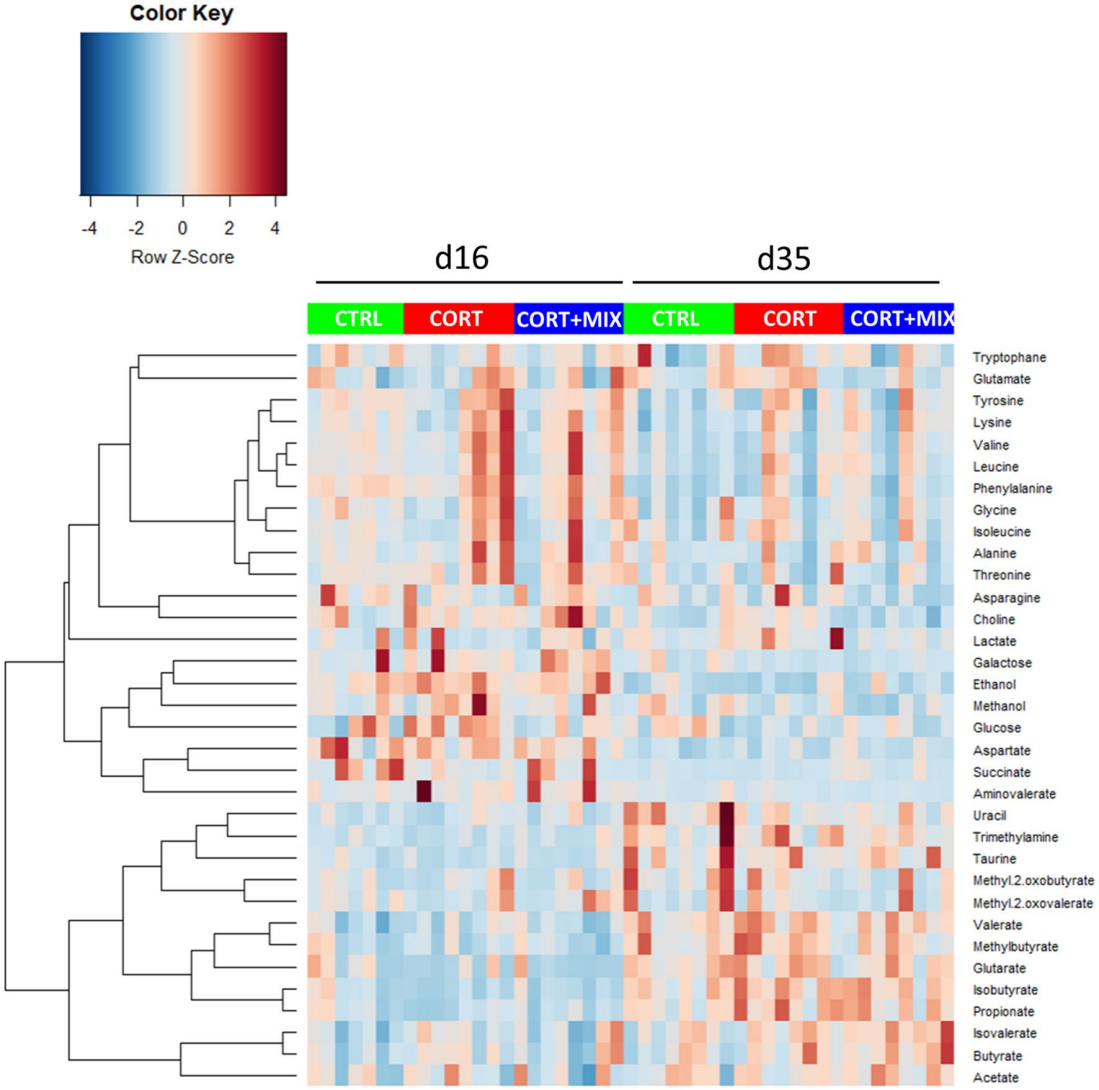
Overview of caecal metabolites variations between individuals. Caecal metabolites relative concentrations were obtained by NMR spectroscopy. Data were transformed by Z-score. The blue color represents the low values while red represents the high values. *CTRL* Control group, *CORT* Birds challenged, *CORT* + *MIX* Birds challenged and supplemented with an amino-acid and polyphenols solution.

At d35, the relative abundance of bacteria belonging to the genus *Roseburia* was significantly lower in the CORT group compared to the CTRL (0.06% versus 0.25%; *p* = 0.01) while its abundance was intermediate in the CORT + MIX group (0.08%). Other genera remained unaffected by the dietary treatments at d16 and d35. Thirty-four metabolites were identified from the caecal content, including short-chain fatty acids, branched-chain fatty acids, AA and oses. While the age was a strong modulator of metabolite relative concentrations no differential caecal metabolic profiles were highlighted between the three groups (Fig. 5).

## Discussion

To elucidate the mechanism of action of the MIX containing functional AA and polyphenols from grape extract, this study investigated the gut health balance of broilers under a physiological challenge, with an emphasis on the intestinal mucosa, the mucus and the microbiota.

Corticosterone is produced via the activation of the hypothalamic–pituitary–adrenal (HPA) axis and is considered as a classic endocrine response of birds to cope with a wide range of stressors. Environmental and social stressors as well as infections and infestations can stimulate its secretion by the adrenal cortex^50^. In return, glucocorticoids modulate the gut paracellular permeability by triggering epithelial cytoskeleton contraction, as shown in rats^51^. In our study, FITC-d permeability assay, optimized for broiler model^41,52^, was used as a direct in vivo evaluation of gut barrier integrity. As expected, we observed an increase of gut paracellular permeability after the administration in drinking water of exogeneous corticosterone in non-supplemented animals^26,51^. The analysis of FITC-d measurement variance revealed different individuals’ sensitivity to CORT, especially when the birds received a standard diet, possibly because of different drinking frequency between the birds resulting to different CORT intake, as previously noticed in quails^33^. The disruption of the physical gut barrier in the challenged and non-supplemented birds of the CORT group is associated to a downregulation of genes coding for TJ proteins like *ZO-1* and *OCLN* and as well as gene coding for Mucin 2, the oligomeric Mucus/Gel-Forming (MUC2). Interestingly, these dysregulations occurred either three days after the stress (d16), a delay corresponding to the time of epithelial cell turnover^53^, or three weeks afterwards (d35) suggesting an imprinting effect of the CORT stress. Such physiological regulations are common features of “leaky gut”^7,54^. Knowing the immunosuppressive effect of CORT on broilers^9^, a lower gene expression of TNFα cytokine in the jejunum of broilers fed a standard diet and stimulated with CORT was expected. In our study, this phenomenon was observed as a trend and should be confirmed by increasing the sample size. We also saw that CORT administration induced adverse effects on crypt structure at d35, suggesting an impairment of the gut epithelium renewal^53^. Overall, the CORT treatment was found relevant to induce a mild stress in broilers.

The supplementation of AA and grape extracts suppressed the long-term adverse effects of CORT on intestinal permeability as indicated by a decrease of plasma FITC-d few days after CORT administration. Besides, the normalization of the gut paracellular permeability, MIX supplementation seems to reduce the FITC-d plasma variability. The gut paracellular permeability is primarily regulated by tight junctions including zonula occludens, occludins and the claudins that seal epithelial cells^55,56^. Surprisingly, the positive effect of the MIX supplementation was not related to modulations of *OCLN* and *ZO-1* jejunum gene expression. It should be however noted that these markers were only evaluated in one part of the digestive tract while gut permeability is the result of all the intestinal segments integrity^56^. Besides, tight junction proteins undergoes constant remodeling and our approach might be to static to highlight transcription regulations of these proteins^57^. Our study provides a variation on Barekatain et al. experiment with synthetic glucocorticoid used instead of corticosterone on broilers supplemented with L-Arg, L-Thr, L-Gln and GSE^26^. When investigating claudins and ZOs levels in the ileum, no changes of transcriptional expression were observed on challenged and supplemented broilers, lending support to our results. The tightness of the epithelial lining is following a dynamic and multi-molecular process with the involvement of TJ but also adherens junctions and desmosomes^7^. A transversal gene expression panel related to intestinal health (genes for barrier function, immune response, oxidation, digestive hormones…) could be used in the future to better capture this complexity, as demonstrated by Criado-Mesas et al.^58^. The maintenance of the gut physical barrier also relies on an appropriate cell turnover. Indeed, the mucosal repair process generally occurs in three phases with first being the mobilization of non-proliferative cells, and in some cases apoptosis, followed by a proliferative burst to renew the lost tissue before a final return to homeostasis^59^. Cells proliferation mainly occurs in the crypts while the villus represents the absorptive surface of the intestines^60^. The changes of gut morphology observed with the AA and GSE supplementation, i.e., a reduction of villus length and surface after the CORT supplementation together with a restoration of crypt depth in the finisher phase, could indicate higher epithelium proliferative activity with the supplementation^25^.

The mucus layer is composed of highly glycosylated proteins called mucins that act as a protective layer on the epithelial cell lines, thus strengthening the physical and chemical gut barrier^61^. In the small intestine, the mucus lining mainly consists of the mucin-2^60^. The addition of AA with polyphenols in the diet seemed to restore the secretion of mucin-2 as indicated by an up-regulation of MUC2 gene expression shortly after the CORT challenge. This effect could be partly related with the L-Thr supplementation, as this AA is directly involved in mucin secretion through regulations of MUC2 transcription^31,62^. Nevertheless, we noted a depletion of goblet cells in the jejunum villi (raw counts or normalized by villous size) of broilers receiving the amino-acid and polyphenols solution after stress induction. This finding should be interpreted with care since the density of goblet cells is not a quantification for mucin secretion (each goblet cell can exhibit different secretory capacity)^36^. Functional AA and polyphenols share similar benefits on the gut health, resulting in possible additive effects when used in combination. For instance, GSE and Thr can both contribute to the mucus defense of the birds^63^.

Knowing the key role of the glucocorticoids in the bidirectional signaling between the gut microbiota and the brain^5^, shifts of the gut microbiome composition was expected after CORT administration. However, the response of the gut microbiota to glucocorticoid is not consistent between animal models, depending on the magnitude of glucocorticoid changes as well as ecological and host factors^64^. In the present trial, the induction of a physiological stress did not modify the caecal microbiota profile and activity. Interestingly, in an adult squirrel model subjected to glucocorticoids variation, hormone variations were mainly affecting rare taxa (< 0.01% of relative abundances) while abundant taxa retained their position by occupying core niches^64^. Such findings should however be taken cautiously knowing the challenging quantification of rare OTUs^65^.

Polyphenols and Arg can both exhibit antimicrobial activities^16,23^, while Gln contributes to oxidative defense as reported in polyphenols^16,66^. Besides these independent benefits, previous evidence suggests that polyphenols could positively interact with AA intestinal functions by targeting the large intestine. Indeed, polyphenols are poorly absorbed in the small intestine and therefore represent substrates available to the lower tract of the intestine^23,67^. Specific phenolic monomers resulting from tannins fermentation by the gut microbiota were thus found in excreta of broiler chickens^23^ and in the blood of broiler hens^63^ receiving grape extracts. Similarly, the administration of a blend of AA with GSE was recently found to affect the piglet gut microbiota metabolism^28^. The possibility of condensed polyphenols from grape extract to reach the lower part of intestine (as reported in a rat model^68^) and to bind specifically to arginine through hydrogen bonds^69^ suggest that the supplementation of the combination of grape extract and Arg could potentiate the effect of Arg in the large intestine. Knowing the ability of tannins to bind to organic N compounds, it can be suggested that dietary polyphenols coat AA and act as a carrier^69^, thus allowing an adequate release in the lower gastrointestinal tract. Therefore, taken together, these data suggest that polyphenols plus AA could contribute to protect the latter, either in a physical way and/or by modulating the gut microbiota activities. Since our findings did not show strong modulations of the gut microbiota with GSE, this latter hypothesis would need further investigation. When carvacrol, a monoterpene phenol, is provided in the feed of broilers infected with *Campylobacter jejuni*, an increase of pathways involved in the biosynthesis of antimicrobial synthesis and bacteriocin was observed, based on inference analysis from16S genes in the caeca^24^. On the contrary, pathways involved in AA degradation were downregulated in broilers supplemented with carvacrol. A similar experimental dysbiosis model would be appropriate to investigate the role of the gut microbiota, mediated by polyphenols, in AA protection.

## Conclusion

The administration of AA together with polyphenols was shown to protect birds from corticosterone challenge, especially the long-term adverse effect on the gut barrier. The bioactive compounds tested seemed to directly act on the intestinal mucosa functions barrier, by stimulating wound healing process such as restoration of crypt depth, enhancing mucus gel secretion and by normalizing pro-inflammatory cytokine gene expression levels.

### Supplementary Information

Supplementary Information.

## Data Availability

Sequencing reads generated and analyzed during the current study are available in the Sequence Read Archive (SRA) repository, Accession Number PRJNA1045378. All the other data generated or analyzed during this study are included in this published article and its supplementary information files. The raw data is available on request from the corresponding author.
