# Supplementary material for: Effect of diet supplemented with functional amino acids and polyphenols on gut health in broilers subjected to a corticosterone-induced stress
Source: Sci Rep. 2024 Jan 10;14:1032. doi: 10.1038/s41598-023-50852-4 (PMC10781708; doi:10.1038/s41598-023-50852-4)
Supplement: Supplementary file 1 — Supplementary Information. [file 41598_2023_50852_MOESM1_ESM.docx]

**Supplemental data**

**Supplemental Table 1**: Sequences of primers used for quantitative real-time PCR

| **Gene** | **Primer Sequence (5’→3’)** | **Accession number** |
| --- | --- | --- |
| *IL-10* | F – CGCTGTCACCGCTTCTTCA  R – TCCCGTTCTCATCCATCTTCTC | NM_001004414.2 |
| *IFNγ* | F – ACACTGACAAGTCAAAGCCGC  R – AGTCGTTCATCGGGAGCTTG | HQ739082.1 |
| *TNFα* | F – GAGCGTTGACTTGGCTGTC  R – GCTGCACATACACAGTCTGA | XM_040647309.1 |
| *OCLN* | F – GAGCCCAGACTACCAAAGCAA  R – GCTTGATGTGGAAGAGCTTGTTG | NM_205128.1 |
| *TJP1* | F – CCGCAGTCGTTCACGATCT  R – GGAGAATGTCTGGAATGGTCTGA | XM_040680632.1 |
| *MUC2* | F – GCCTGCCCAGGAAATCAAG  R – CGACAAGTTTGCTGGCACAT | [XM_040673077.1](https://www.ncbi.nlm.nih.gov/nucleotide/XM_040673077.1?report=genbank&log$=nucltop&blast_rank=6&RID=GJFH98TU013) |
| *GAPDH* | F – GGTGAAAGTCGGAGTCAACGG  R – TCGATGAAGGGATCATTGATGGC | [NM_204305.1](https://www.ncbi.nlm.nih.gov/nucleotide/NM_204305.1?report=genbank&log$=nucltop&blast_rank=13&RID=GJTKRVKZ01R) |
| *β-ACTIN* | F – CAACACAGTGCTGTCTGGTGGTA  R – ATCGTACTCCTGCTTGCTGATCC | X00182 |

**Supplemental Table 2**: Metabolites identified by NMR in the caecal content. For each metabolite, the chemical shifts (δ^1^H) of the main peaks are listed. *: indicates the peak used for quantification.

| **Metabolite** | **δ^1^H (ppm)** |
| --- | --- |
| 2-methylbutyrate | 0.86 (t)* |
| Valerate | 0.89 (t), 1.31 (m)*, 1.53 (m) |
| Butyrate | 0.90 (t)*, 1.56 (m), 2.16 (m) |
| Isovalerate | 0.92 (d)* |
| Isoleucine | 0.94 (t)*, 1.01 (d) |
| Leucine | 0.97 (t)* |
| Valine | 1.00 (d), 1.05 (d)* |
| Propionate | 1.06 (t)*, 2.19 (m) |
| Isobutyrate | 1.07 (d)* |
| 3-methyl-2-oxovalerate | 1.10 (d)* |
| 3-methyl-2-oxobutyrate | 1.13 (d)* |
| Ethanol | 1.19 (t)*, 3.66 (m) |
| Lactate | 1.33 (d)* |
| Threonine | 1.34 (d)* |
| Alanine | 1.48 (d)* |
| 5-aminovalerate | 1.66 (m), 2.24 (t)*, 3.02 (t) |
| Lysine | 1.73 (m)*, 3.03 (t) |
| Glutarate | 1.79 (t)* |
| Acetate | 1.92 (s)* |
| Glutamate | 2.06 (m), 2.13 (m), 2.36 (m)* |
| Succinate | 2.41 (s)* |
| Aspartate | 2.68 (d), 2.71 (d)*, 2.80 (d), 2.83 (d) |
| Asparagine | 2.87 (d), 2.89 (d), 2.95 (d)*, 2.98 (d) |
| Trimethylamine | 2.88 (s)* |
| Choline | 3.20 (s)* |
| Glucose | 5.24 (d)* |
| Galactose | 5.28 (d)* |
| Methanol | 3.36 (s)* |
| Glycine | 3.57 (s)* |
| Taurine | 3.28 (t)*, 3.43 (t) |
| Uracil | 5.81 (d)*, 7.55 (d) |
| Tyrosine | 6.91 (d)*, 7.20 (d) |
| Phenylalanine | 7.34 (d), 7.38 (t), 7.43 (t)* |
| Tryptophane | 7.74 (d)* |

**Supplemental Table 3**: Growth performance of broilers fed experimental diets

| **Performances** | **CTRL** | **CORT** | **CORT+MIX** | **SEM** | ***P*-value^1^** |
| --- | --- | --- | --- | --- | --- |
| *Period d1 to d10* | | | | | |
| n (birds) | 36 | 35 | 33 |  |  |
| BWd10 (g) | 271.8a | 231.0b | 267.1a | 4.2 | <0.001 |
| ADG^1^ (g/d) | 23.2 | 19.2 | 22.7 | 0.9 | 0.38 |
| ADFI^2^ (g/j) | 26.1 | 22.9 | 23.7 | 0.5 | 0.18 |
|  |  |  |  |  |  |
| *Period d10 to d23* | | | | | |
| n (birds) | 28 | 27 | 22 |  |  |
| BWd23 (g) | 1070.5 | 1031.0 | 1003.5 | 15.3 | 0.21 |
| ADG^1^ (g/d) | 61.4 | 61.5 | 56.1 | 1.5 | 0.44 |
| ADFI^2^ (g/j) | 83.6 | 81.9 | 74.7 | 1.7 | 0.50 |
|  |  |  |  |  |  |
| *Period d23 to d35* | | | | | |
| n (birds) | 28 | 27 | 21 |  |  |
| BWd35 (g) | 2143.4 | 2258.7.0 | 2231.8 | 34.7 | 0.31 |
| ADG^1^ (g/d) | 82.5 | 94.3 | 93.3 | 2.8 | 0.27 |
| ADFI^2^ (g/j) | 134.0 | 136.4 | 124.2 | 3.5 | 0.31 |
|  |  |  |  |  |  |
| *Period d0 to d35* | | | | | |
| ADG^1^ (g/d) | 58.4 | 61.6 | 60.2 | 0.8 | 0.27 |
| ADFI^2^ (g/j) | 81.3 | 80.0 | 70.6 | 1.7 | 0.16 |

^1^Average daily gain

^2^Average daily feed intake. Feed intake was recorded per cage (n=4).
 ^a,b^ values within a line with different superscripts differ significantly at *P*<0.05.
